# Fast high-resolution lifetime image reconstruction for positron lifetime tomography

**DOI:** 10.1038/s42005-025-02100-6

**Published:** 2025-04-26

**Authors:** Bangyan Huang, Zipai Wang, Xinjie Zeng, Amir H. Goldan, Jinyi Qi

**Affiliations:** 1 Departement of Biomedical Engineering, University of California, 451 Health Sciences Dr., Davis, CA USA; 2 Departement of Radiology, Weill Cornell Medicine, Cornell University, 520 East 70th Street, New York City, NY USA

**Keywords:** Computational science, Biophotonics

## Abstract

Due to the ortho-positronium formed prior to the annihilation, the lifetime of a positron is sensitive to the tissue microenvironment and can potentially provide valuable information for monitoring disease progression and treatment response. However, the lifetime of positrons before annihilation has long been overlooked in current positron emission tomography (PET). Here we develop a positron lifetime image reconstruction method called SIMPLE (Statistical IMage reconstruction of Positron Lifetime via time-wEighting) and demonstrate its performance using a real scan on a time-of-flight PET scanner. The SIMPLE method achieves high-resolution positron lifetime imaging of extended heterogeneous tissue illuminated by a ^22^Na point source, successfully resolving the boundary between muscle and fat. It delivers spatial resolution comparable to that of conventional PET activity images while maintaining a computational cost equivalent to reconstructing two PET images. This work paves the way for clinical translation of high-resolution positron lifetime imaging.

## Abstract (web summary)

Current positron emission tomography (PET) techniques overlook the life history of positrons before annihilation, missing potential insights into tissue microenvironments. Here, the authors introduce SIMPLE, an image reconstruction method, achieving high-resolution positron lifetime imaging on a time-of-flight PET scanner, offering new avenues for disease monitoring and treatment assessment.

## Introduction

Positron emission tomography (PET) is a highly effective medical imaging modality that enables the quantification of molecular-level activities within a living body. To achieve this, radiotracers are injected into human bodies and engage in physiologic or biochemical processes. The spatiotemporal distribution of the radiotracer is obtained by detecting coincident 511-keV photons produced by positron annihilations and image reconstruction mostly by maximum-likelihood (ML)-based algorithms.

The life history of positrons is completely ignored by current PET techniques. Due to the presence of positronium, a bound state of an electron and a positron formed before approximately 40% of annihilations in a human body^1^, the positron annihilation can be noticeably delayed relative to the time of positron generation. There are two types of positronium, ortho-positronium (o-Ps) and para-positronium (p-Ps), with o-Ps occurring 75% of the time and p-Ps occurring 25% of the time. The lifetime of p-Ps is relatively short^2^ (125 ps) and is unlikely to be affected by the surrounding microenvironment due to its large annihilation rate. The annihilation of o-Ps in vacuum is very slow with a lifetime of 142 ns^3^. However, in biological tissue the o-Ps lifetime can be reduced to from 1.8 ns^4^ (lifetime in pure water) to 4 ns^5^ (as measured for human skin) due to two effects^6^ associated with the surrounding microenvironment: pick-off annihilation and spin-exchange interaction. Pick-off annihilation occurs when the positron of the o-Ps annihilates with a foreign electron, while spin-exchange is induced when the surrounding molecules possess unpaired electrons. Therefore, the o-Ps lifetime is dependent on the size of intermolecular voids and the concentration of bio-active molecules in biological materials^7^. Studies have revealed the lifetime variation induced by these two factors. Oxygen is one of the molecules affecting the o-Ps lifetime. In a recent study using positron annihilation lifetime spectroscopy (PALS)^8^, the annihilation rate of o-Ps in water is shown to be linearly proportional to the dissolved oxygen concentration (pO_2_). This finding indicates the potential of o-Ps lifetime in identifying hypoxia, and therefore providing useful information for disease progression and treatment response^9,10^. Large lifetime contrast has also been observed between tissues with distinct microstructures. The positronium lifetime image^11^ of spatially separated cardiac myxoma samples and adipose samples showed that the o-Ps lifetimes in cardiac myxoma tissue were 1.950 ± 0.019 ns and 1.874 ± 0.020 ns for two subjects, respectively, and the o-Ps lifetimes in adipose were 2.645 ± 0.027 ns and 2.581 ± 0.030 ns for the same two subjects, respectively.

Performing positronium lifetime imaging (PLI) using time-of-flight (TOF) PET scanners is a promising approach to investigate positronium lifetime in vivo. Several challenges of in vivo PLI have been addressed by recent advances in PET imaging. The first one is radioisotopes with a prompt gamma emission to mark the time of positron emission. ^44^Sc can be an ideal radionuclide for PLI because of its high production rate for prompt gamma and distinguishable prompt-gamma energy as compared to 511 keV. Two human PET scans^12,13^ using ^44^Sc-labeled DOTATOC and PSMA-617 have been reported. Meanwhile, several other radionuclides that have already been widely used in PET, such as ^124^I^14^, ^82^Rb^15^, and ^68^Ga^16^, could also be candidates for PLI. The second challenge is high efficiency for detecting lifetime events (known as triple coincidences), each comprising of a prompt gamma together with the associated annihilation photon pair. This has been addressed by the development of long axial field-of-view (FOV) scanners^17–20^. Computer simulation shows the sensitivity of triple coincidence detection on the EXPLORER scanner is even higher than the detection of 511-keV pairs on current whole-body PET scanners^21,22^.

The remaining challenge is the need for high-resolution lifetime image reconstruction methods. An existing reconstruction method for o-Ps lifetime is to use the TOF information to localize each PLI event at the most likely position along the line-of-response (LOR) and fit the lifetime spectrum of the events in each voxel to form the lifetime image^11,21^, which is referred to as the direct TOF-backprojection (TOF-BP). The direct TOF-BP method suffers from low resolution and high noise. To obtain high-resolution lifetime images, we have introduced a penalized maximum likelihood (PML) method^23^. This method can produce high-resolution lifetime images but suffers from high computational cost and uses a mono-exponential decay model that is inadequate for real-world lifetime distributions. Another method that we developed^24^, known as SPLIT (Statistical Positronium Lifetime Image reconstruction via time-Thresholding), can perform 3D reconstruction and correct random events. This method leverages existing activity reconstruction algorithms, such as ordered-subset expectation-maximization (OSEM), to reconstruct a threshold-activity curve for each voxel and then estimate the lifetime image from these curves. While it has much lower computational cost than the PML method, it still requires reconstruction of tens of activity images to form the threshold-activity curves and perform curve fitting for each voxel. At a low count level, which could be the common case for PLI, the fitting of the threshold-activity curves can be unstable. In this paper, we focus on the average lifetime, referred to as positron lifetime, of all interaction pathways including direct annihilation and positronium formation. We propose a simple yet effective positron lifetime reconstruction method that is more efficient and robust than the previous methods. The proposed method is called SIMPLE, which stands for Statistical IMage reconstruction of Positron Lifetime via time-wEighting.

Furthermore, most existing PLI studies were performed using a ^22^Na point source that is directly embedded into the tissue sample^11^. This design leads to the effective region of interest (ROI) being basically a point as the result of the limited positron range. It also mixes the interested lifetime events from the tissue with the events originated in the covering material of the source (usually Kapton foil), which complicates the analysis of tissue lifetime. To achieve high-resolution PLI on extended ex vivo tissue samples, we designed a PLI experiment by suspending a ^22^Na source in air to illuminate positrons to an extended area. Subsequently, we applied the proposed SIMPLE method to demonstrate its capability of obtaining high-resolution positron lifetime images.

## Results

### Simulated small-animal scan

We performed a Monte Carlo simulation to validate the SIMPLE method. A rodent phantom was simulated in GATE^25^ with an average activity concentration of 20 kBq/cc and a scan duration of 30 minutes. A spherical lesion with a diameter of 10 mm was inserted into the lower right flank of the body. The activity concentration ratio was set to 10 : 15 : 2 : 1 for the lesion, kidneys, liver, and body background, respectively, mimicking ^44^Sc-PSMA uptake. A three-component lifetime distribution was simulated. The intensities of the o-Ps, p-Ps and direct annihilation were 30%, 10%, and 60%, respectively, and were kept uniform within the object. The o-Ps lifetime was set to 2.0 ns inside the lesion and 2.5 ns elsewhere; the lifetimes of p-Ps and direct annihilation were set to 0.125 ns and 0.4 ns, respectively, throughout the object. Thus, the average lifetime was 0.8525 ns for the lesion and 1.0025 ns elsewhere. The simulated PET scanner was the Neuro-EXPLORER scanner^20^ with a ring diameter of 52 cm, an axial length of 49 cm, and a TOF resolution of 250 ps. The energy resolution was set to 13% at 511 keV.

The triple coincidences were formed based on the single events recorded. Every triple coincidence with two annihilation photons less than 1 ns away in the detection time and with a lifetime measurement from –20 ns to 20 ns was identified and recorded. The energy window for 511-keV photons was [430, 650] keV. For the 1157-keV prompt gammas, the lower energy threshold was 700 keV and there was no higher energy threshold. The travel time difference was corrected for each PLI event prior to the reconstruction.

**Fig. 1:**
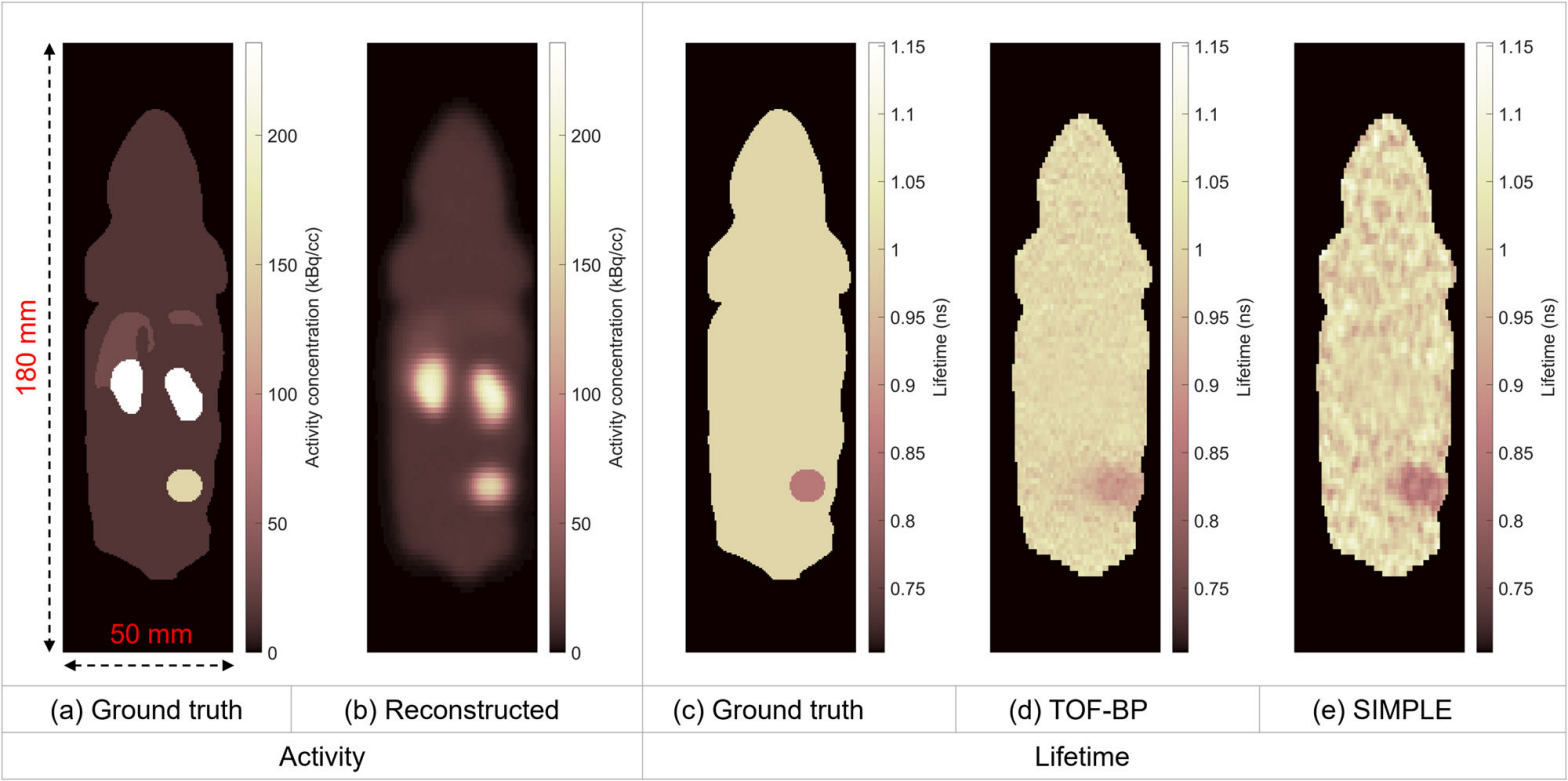
Reconstructed activity and lifetime images of the phantom. **a** The ground truth of activity concentration. **b** The reconstructed activity image using the 511-keV photons in the triple coincidences. **c** The ground truth of lifetime image, where the simulated tumor has lower lifetime than normal tissue. **d** The reconstructed lifetime image by the direct TOF-BP method. **e** The reconstructed lifetime image by the SIMPLE method, showing sharper tumor boundary than the TOF-BP image.

A total of 94.0 M triple coincidences were obtained in the reconstruction time window from –1 to 15 ns ($[{t}_{{{\rm{r}}}1},{t}_{{{\rm{r}}}2}]$ in Eq. (14)) and 4.1 M triple coincidences were obtained in the correction time window from –20 to –5 ns ($[{t}_{{{\rm{c}}}1},{t}_{{{\rm{c}}}2}]$ in Eq. (14)). The reconstructed activity (from 511-keV coincidence pairs in the triples) and lifetime images are shown in Fig. 1. The activity image shown is at the same iteration as the SIMPLE image. The positron lifetime in different ROIs is listed in Table 1. Note that when displaying the lifetime image, a voxel is set to zero if its activity is below 50% of the intensity in the body background. The direct TOF-BP can reconstruct accurate average lifetime for the large background region with a constant lifetime value, but the lifetime value in the lesion was significantly overestimated due to the low spatial resolution of the direct TOF-BP. The proposed SIMPLE method shows accurate average lifetime in both lesion and normal regions. The standard deviation (s.d.) of the voxel values in the lifetime image is well controlled as compared to the 0.15-ns contrast between the lesion and normal regions.

**Table 1:** Quantification of lifetime images of the phantom

	Lesion	Kidney	Liver	Body background
Relative Activity concentration	10	15	2	1
Ground truth lifetime (ns)	0.8525	1.0025	1.0025	1.0025
Direct TOF-BP (ns)	0.906 ± 0.012	1.004 ± 0.009	1.006 ± 0.013	1.001 ± 0.021
SIMPLE (ns)	0.863 ± 0.020	0.997 ± 0.017	1.001 ± 0.029	0.999 ± 0.041

Mean ± standard deviation of the voxel value in different ROIs of the images reconstructed by the direct TOF-BP and SIMPLE method.

The positron lifetime image is evaluated against the o-Ps lifetime image reconstructed by the SPLIT method^24^. Since we used the same settings for the OSEM reconstruction as those in the SPLIT reconstruction (see Methods section for details), the two reconstructions achieve similar spatial resolution. However, the noise in the SIMPLE image is noticeably lower than that in the SPLIT image. This improvement can be quantified using the signal-to-noise ratio (SNR), defined as:

1 $${{\rm{SNR}}}=\frac{{{\rm{|lesion}}}-{{\rm{background|}}}}{{{\rm{background}}}\; {{\rm{standard}}}\; {{\rm{deviation}}}}$$

where the signal is the difference between the ROI means in the lesion and background regions, while noise is calculated as the standard deviation of voxel values in the body background ROI. The SIMPLE method achieved a SNR of 3.3, outperforming the SPLIT image, which had an SNR of 2.5.

### The real experimental scan

**Fig. 2:**
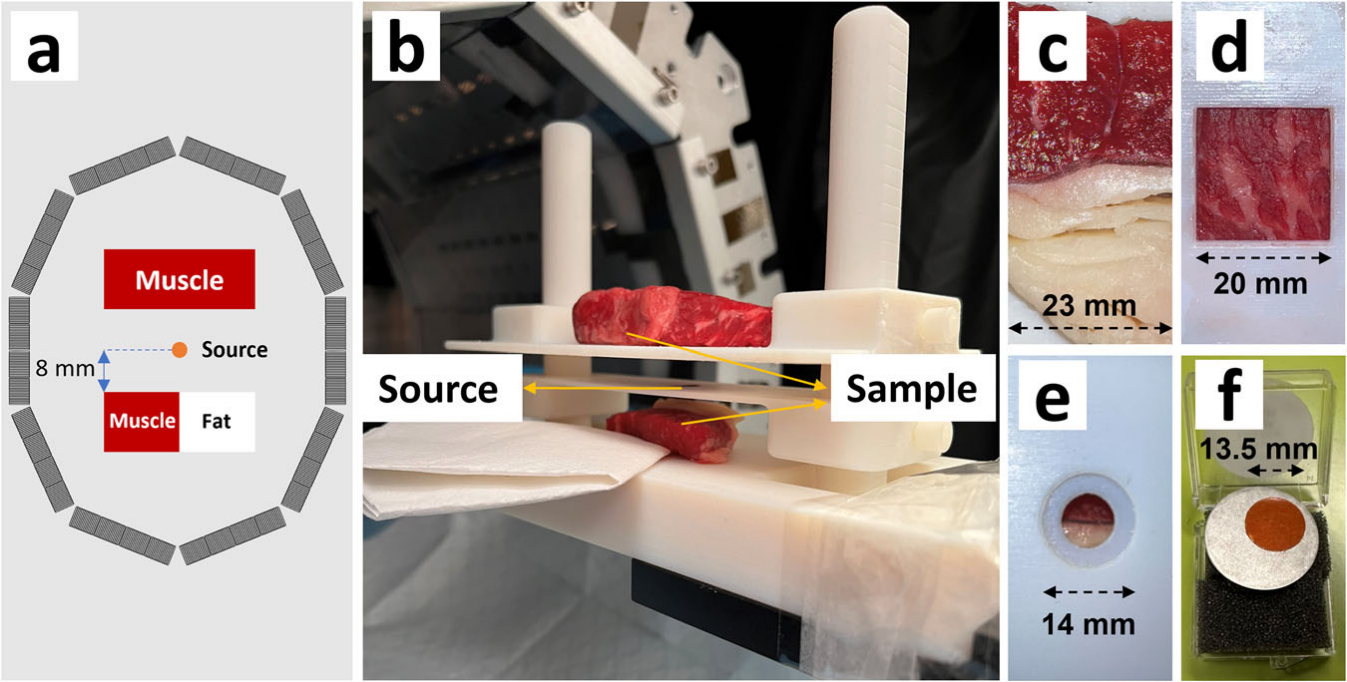
The experimental scan setup. **a** Schematic illustration of the experimental setup of the source and biological tissue, which are placed within the FOV of the Prism-PET scanner. The point source is suspended over the sample with an 8-mm clearance. **b** The 3D-printed holder with the source and sample. **c** The bottom beef sample consists of muscle and fat tissue. **d** The top beef sample looked from bottom. The fat content is visible. **e** The tray for the ^22^Na source looked from above. Positrons pass through the hole and irradiate the bottom sample. **f** The ^22^Na point source (orange disk).

Here we present an experiment to realize high-resolution lifetime imaging of an extended area on the surface of a sample. The design is to utilize the positron range in the air to irradiate the tissue sample with free positrons so that positron annihilation can occur in an extended area, thereby enabling lifetime imaging on a heterogeneous sample (Fig. 2a, b). The ^22^Na point source used is shown in Fig. 2f. The sodium source is covered by Kapton foil with a thickness of 0.06 mm, allowing positrons to escape out of the source and become free positrons in the air. To maximize the usage of positrons, we suspended this source in the air on a tray with a circular hole beneath it (Fig. 2e) and placed one biological sample at the top and one at the bottom. We used beef in this experiment. The bottom sample (Fig. 2c) consists of fat and muscle with a clear boundary, which was aligned with the source horizontally. The top one (Fig. 2d) is a muscle tissue but with visible fat content distributed in it. The source was 8 mm away from the bottom sample and 13 mm from the top one. The activity of the ^22^Na point source was 3.7 MBq. We used the Prism-PET prototype scanner^26,27^ to accomplish the scan. The single-ring scanner has a decagon shape with a long diameter of 38.5 cm, a short diameter of 29.1 cm, and an axial FOV of 25.5 mm. The TOF resolution is 271 ps. During the scan, it recorded prompt double coincidences in a coincidence window of 15 ns using the take-all-good policy with a wide energy window of [370, 1500] keV. No delayed time window was applied. The total scan lasted 13 hours and a total of 333 M double coincidences were collected. The triple coincidences were grouped from the double coincidences. During this process, we identified the 1275-keV prompt gamma event as being over 700 keV and an annihilation photon pair within an energy window of [440, 620] keV. We retained triples with two 511-keV photons closer than 1 ns; the lifetime measurements range from -15 ns to 15 ns, which was determined by the 15-ns coincidence window. A total of 4 M triple coincidences were obtained with an effective sensitivity of 0.0023%. These PLI events were then corrected for the travel distance before the subsequent reconstruction. The reconstruction time window was [–1, 15] ns and the correction time window was [–15, –1] ns.

**Fig. 3:**
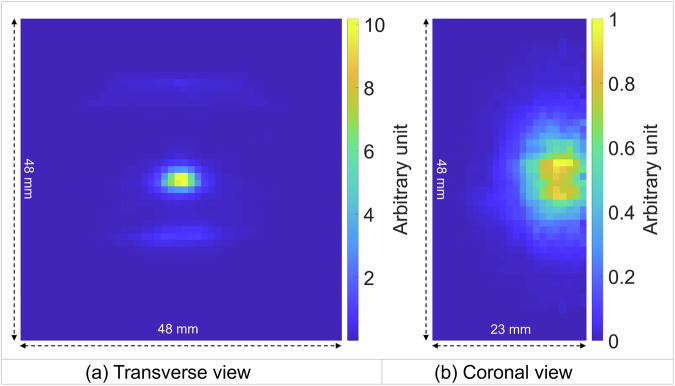
Reconstructed activity image. A transverse and a coronal slice are shown in (**a**) and (**b**), respectively. The bright spot at the center of the transverse slice corresponds to the ^22^Na point source and the two horizontal streaks represent positron annihilations within the tissue samples. The coronal slice is 8.5 mm below the ^22^Na source.

The reconstructed activity image is shown in Fig. 3. From the transverse slice we can see that although the point source still has a high intensity, there is a significant number of annihilations occur in the tissue samples above and below the point source. The coronal slice of the activity image of the bottom fat-and-meat sample shows that the activity intensity gradually decreases as the distance to the source increases and the distribution of the activity is primarily in a circle of ~25-mm diameter. The cut-off on the right-hand side in the coronal images is due to the limited axial FOV.

**Fig. 4:**
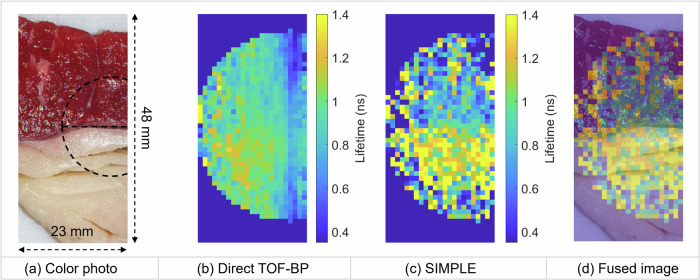
Reconstructed lifetime images of the tissue. **a** Color photo of the tissue sample. The two dashed circular regions (10-mm radius) indicate the muscle and fat ROIs used for lifetime quantification in Table 2. **b** Lifetime image reconstructed by the direct TOF-BP method. The lifetime value is only shown within a circular region where sufficient positron annihilations occur. **c** Lifetime image reconstructed by the SIMPLE method, showing sharp boundary between muscle and fat. **d** Fused image of (**a**) and (**c**).

The coronal slices of the positron lifetime images of the bottom sample are shown in Fig. 4. The boundary between the fat and muscle is indistinct in the direct TOF-BP image, while the SIMPLE method can clearly resolve the lifetime difference between these two types of tissue. The quantitative values of the average lifetime are listed in Table 2. The ROIs are drawn as circular regions with a radius of 10 mm and a thickness of 3 mm; the ROIs in the bottom tissue are illustrated in Fig. 4a as two quarter circles. In the bottom tissue, the average lifetime measured by the SIMPLE method is 0.88 ns for the muscle and is 1.23 ns for the fat. The longer positronium lifetime in fat is consistent with the results previously published^11,28^. In comparison, the average lifetimes measured by the direct TOF-BP are 0.86 ns and 0.95 ns for the muscle and fat, respectively. The reason that the direct TOF-BP yields a shorter average lifetime compared to the SIMPLE method is because the annihilations originated inside the Kapton foil have a much shorter lifetime^29^ and the direct TOF-BP method misplaces those events to the tissue sample. Comparing the lifetime values in the bottom and top muscle tissues, we can observe that the average lifetime is longer in the top one. This is expected as the top muscle sample is fattier as compared to the bottom one.

**Table 2:** Quantification of reconstructed lifetime

		dTOF-BP	SIMPLE
Top	Muscle (ns)	0.86 ± 0.09	0.96 ± 0.33
Bottom	Muscle (ns)	0.86 ± 0.11	0.88 ± 0.34
	Fat (ns)	0.95 ± 0.12	1.23 ± 0.29

Mean ± standard deviation of the voxel value is evaluated for each ROI.

## Discussion

We propose a fast and yet effective positron lifetime image reconstruction method and demonstrate high-resolution positron lifetime imaging on an extended heterogeneous sample with a simple yet effective experiment design. The average positron lifetime can serve as a biomarker as effectively as the more extensively studied o-Ps lifetime. It has been shown that the o-Ps lifetime not only has a longer lifetime in adipose tissue but is also of a higher intensity^28^ (${A}_{o}$ in Eq. (2)). Thus, a higher contrast could be reflected by the positron lifetime than by the o-Ps lifetime. Furthermore, the reconstruction of positron lifetime using the SIMPLE method is much easier and more robust than the reconstruction of o-Ps lifetime. The SIMPLE method only requires minimal modification to the standard MLEM algorithm. No exact lifetime model is required for the SIMPLE method, whereas the reconstruction of o-Ps lifetime relies on an accurate lifetime model, whose parameter can be difficult to obtain in real scenario. Comparing the simulation results with those in the SPLIT paper^24^, we found that the average lifetime image provides higher SNR for lesion detection than the o-Ps lifetime image reconstructed by the SPLIT method. This is consistent with the findings reported by Moskal et al.^30^. The experimental design we developed is a simple and effective approach that achieves (1) lifetime imaging in an extended area and (2) removal of the interference from the source material in lifetime measurement. The success of this experiment demonstrates the feasibility of exploiting positron range in the air. In the future, the proposed experimental design can be used to measure the lifetime of other types of tissues.

The finding of the contrast between the fat and muscle tissues is expected. Several other studies^11,28^ also report a longer positronium/positron lifetime in human adipose than in other human tissues. This phenomenon could be due to the distinct microstructure of the adipose tissue. However, the structural differences among other tissues could be more nuanced^31^ than the distinction of adipose tissue, leading to a smaller lifetime contrast. There is also a lack of relevant studies that can provide validation to the obtained lifetime values. Even if such a measurement existed, its results may not constitute a reliable ground truth to the reported experiment because the conditions such as temperature, humidity, and the freshness of the sample may all affect the positron lifetime.

The loss function minimized by the SIMPLE method is the Kullback–Leibler (KL) distance. We adopt this metric because the optimization process can easily be accomplished by a modified MLEM algorithm. Minimizing the KL distance has its meaning in standard PET—to obtain an ML estimator for the activity map. However, the data we constructed for the lifetime-weighted image no longer admit a Poisson distribution, which renders the estimator non-ML. Meanwhile the real data with negative lifetime measurements violate the definition of the KL distance between two vectors with non-negative entries. We have managed to include as few negative lifetime measurements as possible by setting the lower bound of reconstruction time window ${t}_{r1}$ close to zero. This minor violation in practice is proven to be acceptable as the reconstructed lifetime in the simulation scan is accurate. It is worth investigating to use or design other loss functions to achieve a better performance than the KL distance. Another direction to improve the performance is regularization. Currently the SIMPLE method is subject to high noise and only images at early iteration are used, resulting in a degraded spatial resolution. We will investigate applying regularization to either the intermediate activity and lifetime-weighted images or the final lifetime image.

## Methods

### PLI event model

In this paper, a PLI event is defined as a triple coincidence consisting of two annihilation photons and a prompt gamma. It is mathematically represented by a TOF sinogram bin ${i}_{k}$ determined by the LOR and the TOF bin of the coincidence event and a lifetime measurement ${\tau }_{k}$, where $k$ is the list-mode index. The distribution of the lifetime measurement of true PLI events (without random events) can be written as^32,33^:

2 $$p\left(\tau |{{\boldsymbol{\lambda }}},{{\boldsymbol{A}}}\right)=g\left(\tau -\mu \right)* {\sum}_{l\in \left\{o,p,d\right\}}{A}_{l}{\lambda }_{l}\, exp \left(-{\lambda }_{l}\tau \right)u\left(\tau \right)$$

where $u\left(t\right)$ is the unit step function. The subscript $l\in \left\{o,p,d\right\}$ denotes an annihilation pathway corresponding to o-Ps, p-Ps and direct annihilation, respectively. ${A}_{l}$ denotes the intensity of the ${l}^{{th}}$ pathway and ${\lambda }_{l}$ is the annihilation rate (i.e., the inverse of the lifetime). $g\left(\tau -\mu \right)$ is a Gaussian function accounting for the detector timing response, where $\mu$ is the timing offset. The resolution of this function is determined by the detector timing resolution and the residual errors in correcting the travel time of the prompt gamma and the annihilation photon pair before detection.

It is important to emphasize that the goal of the proposed SIMPLE method is not to estimate the o-Ps lifetime but the positron lifetime, which is defined as the average lifetime including all the interaction pathways. The positron lifetime $m$ at voxel $j$ is

3 $${m}_{j}={\int }_{\!\!\!-\infty }^{+\infty }\tau \times p\left(\tau |{{{\boldsymbol{\lambda }}}}_{j},{{{\boldsymbol{A}}}}_{j}\right)d\tau -\mu$$

### Image reconstruction algorithm

Instead of directly reconstructing for positron lifetime image, we reconstruct a lifetime-weighted activity image

4 $${w}_{j}={x}_{j}{m}_{j}$$

where $j$ is voxel index; ${{\boldsymbol{x}}}$ is the activity image; and ${{\boldsymbol{m}}}$ is the positron lifetime image. The lifetime image ${{\boldsymbol{m}}}$ can then be estimated by taking the voxel-wise ratio of ${{\boldsymbol{w}}}$ over ${{\boldsymbol{x}}}$.

To reconstruct the lifetime-weighted activity image ${{\boldsymbol{w}}}$, we need to construct a projection data vector ${{\boldsymbol{z}}}$ whose expectation is the forward projection of ${{\boldsymbol{w}}}$.

5 $${\bar{z}}_{i}={\sum }_{j}^{N}{H}_{{ij}}{w}_{j}={\sum }_{j}^{N}{H}_{{ij}}{x}_{j}{m}_{j}={\sum }_{j}^{N}E\left[{y}_{{ij}}\right]{m}_{j}$$

where ${y}_{{ij}}$ is the number of PLI events originated in voxel $j$ and detected in TOF sinogram bin $i$; $N$ is the number of voxels; and ${{\boldsymbol{H}}}$ is a standard TOF system matrix whose element ${H}_{{ij}}$ denotes the probability of detecting a coincident event originated in voxel $j$ in TOF sinogram bin $i$. Note that we use standard TOF system matrix for all the reconstruction in this paper. For the reconstruction of PLI events, a complete system matrix ${{\boldsymbol{P}}}$ should include the efficiency for detecting the prompt gammas: ${{\boldsymbol{P}}}={{\boldsymbol{HQ}}}$, where ${{\boldsymbol{Q}}}$ is a diagonal matrix with every diagonal element corresponding to the sensitivity for prompt gammas emitted from a voxel and is nontrivial to obtain. For the SIMPLE method, however, it is not necessary to explicitly obtain ${{\boldsymbol{Q}}}$ as its effect on images ${{\boldsymbol{x}}}$ and ${{\boldsymbol{w}}}$ will be canceled out in the lifetime image formed by the element-wise division ${{\boldsymbol{w}}}/{{\boldsymbol{x}}}$. Due to this reason, we omit ${{\boldsymbol{Q}}}$ in all the images reconstructed using ${{\boldsymbol{H}}}$ in this paper.

Continuing with Eq. (5), we can utilize the fact that ${E[{y}_{{ij}}]m}_{j}=E[{\sum}_{k\in {K}_{{ij}}}{\tau }_{k}],$ where ${K}_{{ij}}$ denotes the set of list-mode indices of the events originated in voxel $j$ and detected in bin $i$. Then we get

6 $${\bar{z}}_{i}={\sum }_{j}^{N}E\left[{y}_{{ij}}\right]{m}_{j}={\sum }_{j}^{N}E\left[{\sum}_{k\in {K}_{{ij}}}{\tau }_{k}\right]=E\left[{\sum}_{k\in {K}_{i}}{\tau }_{k}\right]$$

where ${K}_{i}={\bigcup}_{j}{K}_{{ij}}$. Equation (6) indicates that the projection data ${{\boldsymbol{z}}}$ can be constructed by summing the lifetime measurements in each TOF sinogram bin:

7 $${z}_{i}={\sum}_{k\in {K}_{i}}{\tau }_{k}$$

The lifetime-weighted image can be obtained by minimizing the KL distance^34^ between the data vector ${{\boldsymbol{z}}}$ and the forward projection ${{\boldsymbol{Hw}}}$

8 $$\hat{{{\boldsymbol{w}}}}={{{\rm{argmin}}}}_{{{\boldsymbol{w}}}}{KL}\left({{\boldsymbol{z}}},{{\boldsymbol{Hw}}}\right)$$

The KL distance is defined between two vectors ${{\boldsymbol{a}}}$ and ${{\boldsymbol{b}}}$ with nonnegative entries: ${KL}\left({{\boldsymbol{a}}},{{\boldsymbol{b}}}\right)={\sum}_{i}{KL}({a}_{i},{b}_{i})$. For ${a}_{i} > 0$ and ${b}_{i} > 0$, ${KL}\left({a}_{i},{b}_{i}\right)={a}_{i}\log \left(\frac{{a}_{i}}{{b}_{i}}\right)+{b}_{i}-{a}_{i}$; ${KL}\left({a}_{i},0\right)=+\infty$; and ${KL}\left(0,{b}_{i}\right)={b}_{i}$. Using the optimization transfer (OT) principle, one can obtain a surrogate function via the concavity of logarithm function

9 $$\phi \left({{\boldsymbol{w}}};{{{\boldsymbol{w}}}}^{n}\right) = {\sum }_{i=1}^{M}\left[-{z}_{i}{\sum }_{j=1}^{N}\frac{{H}_{{ij}}{w}_{j}^{n}}{{\sum }_{l=1}^{N}{H}_{{il}}{w}_{l}^{n}}\log ({w}_{j})+\mathop{\sum }_{j=1}^{N}{H}_{{ij}}{w}_{j}\right]$$

where $n$ is the $n$-th step of update and $M$ is the total number of TOF sinogram bins. Taking the derivative of the surrogate function, the update ${{{\boldsymbol{w}}}}^{n+1}$ that minimizes the surrogate function is

10 $${w}_{j}^{n+1}=\frac{{w}_{j}^{n}}{{\sum }_{i=1}^{M}{H}_{{ij}}}\mathop{\sum }_{i=1}^{M}\frac{{H}_{{ij}}{z}_{i}}{{\sum }_{l=1}^{N}{H}_{{il}}{w}_{l}^{n}}$$

To obtain a list-mode update equation, one can substitute Eq. (7) into Eq. (10), resulting in the list-mode update equation as

11 $${w}_{j}^{n+1}=\frac{{w}_{j}^{n}}{\mathop{\sum }_{i=1}^{M}{H}_{{ij}}}\mathop{\sum}_{k}\frac{{H}_{{i}_{k}j}{\tau }_{k}}{{\sum }_{l=1}^{N}{H}_{{i}_{k}l}{w}_{l}^{n}}$$

Equation (11) is very similar to MLEM algorithm with the only difference being the weighting factor ${\tau }_{k}$ for every event.

### Correction for scanner timing offset

The timing offset $\mu$ in Eq. (2) is caused by the different timing responses to prompt gammas and 511-keV photons. Failure to accurately determine this offset will lead to bias in positron lifetime estimation. In the experimental study, we fit the spectrum of all the lifetime measurements to obtain this value. To increase the robustness of the fitting, only two components were assumed in a fitting model

12 $${p}_{1}\left(\tau |{{\boldsymbol{\theta }}}=[{{\boldsymbol{\lambda }}},{{\boldsymbol{A}}},b,G,\mu ]\right) = G \times \left[g\left(\tau -\mu \right)* \mathop{\sum }_{l=1}^{2}{A}_{l}{\lambda }_{l} exp \left(-{\lambda }_{l}\tau \right)u\left(\tau \right)+b\right]$$

where $b$ accounts for the type I randoms (details in the next section); ${A}_{1}+{A}_{2}=1$; and $G$ is a scaling factor to match the intensity of the spectrum. To obtain the parameter $\mu$, we minimize the weighted least squares loss

13 $${{\boldsymbol{\theta }}}={{{\rm{argmin}}}}_{{{\boldsymbol{\theta }}}}{\sum }_{i=1}^{N}\frac{1}{{n}_{i}}{\left({p}_{1}\left({\tau }_{i}|{{\boldsymbol{\theta }}}\right)-{n}_{i}\right)}^{2}$$

where the spectrum is divided into $N$ bins with counts within each bin denoted by ${n}_{i}$ and ${\tau }_{i}$ denotes the center of each histogram bin. No time delay was set in the simulation scan. In the experiment, $\mu$ was determined to be 0.239 ns, which was then subtracted from the reconstructed lifetime image.

### Random events correction

The accuracy of lifetime estimates can be compromised by random events if left uncorrected. In our previous paper^24^, we have given a thorough analysis of the randoms events. Here we briefly review the classification of randoms (Table 3). Based on the criterion whether every two single events in a triple coincidence are from the same decay, the random events can be categorized into three types. Type I randoms are formed by a pair of 511-keV photons from the same annihilation with a random prompt gamma; Type II randoms are formed by a 511-keV photon and a prompt gamma from the same decay with a random 511-keV photon; Type III randoms are formed by photons from three different decays.

**Table 3:** Summary of possible relationships between each two single events of a triple coincidence

		1^st^ 511 – 2^nd^ 511	1^st^ 511 – PG	2^nd^ 511 – PG
True events		T	T	T
Random events	Type I	T	R	R
	Type II.a	R	T	R
	Type II.b	R	R	T
	Type III	R	R	R

T means the two events are from the same decay and R means they are from two different decays (i.e., random). The order of the 1^st^ and the 2^nd^ 511-keV single events is determined by their detected times.

**Fig. 5:**
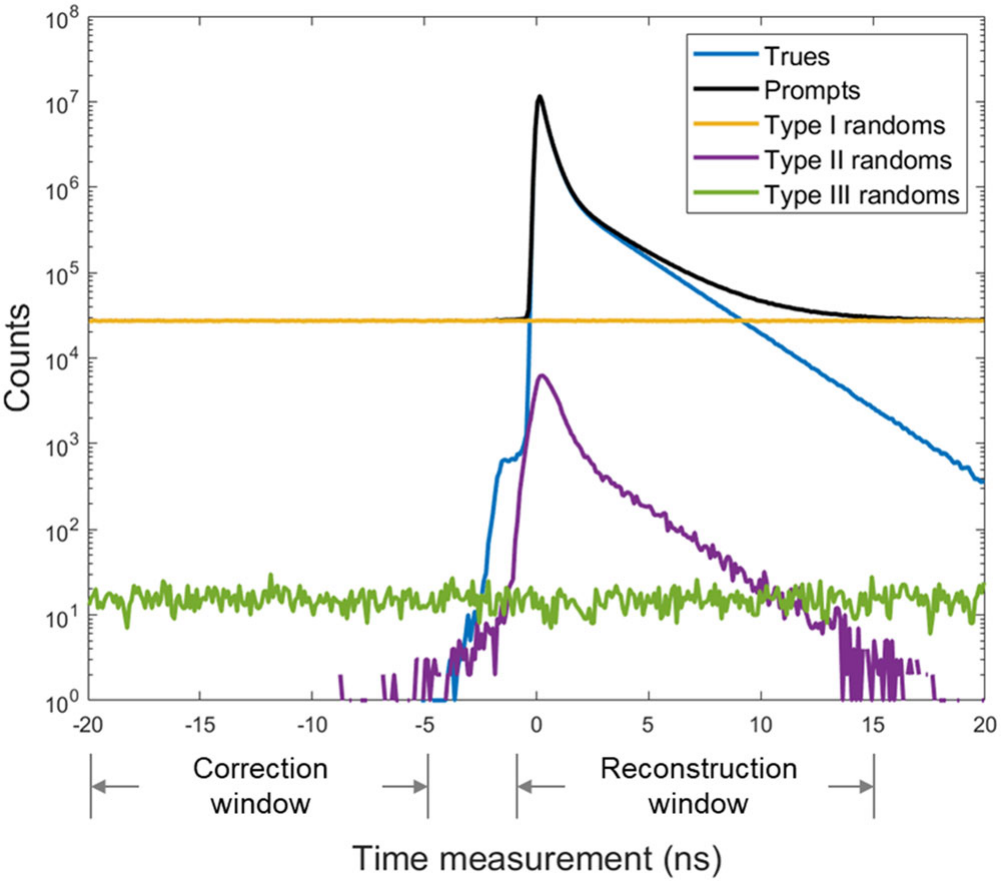
The histogram of the lifetime measurements from the simulation scan. True and randoms events are identified by examining the event ID output by GATE. For the simulation study, the correction and reconstruction time windows are $\left[-20,\,-5\right]{{\rm{ns}}}$ and $\left[-{\mathrm{1,15}}\right]{{\rm{ns}}}$ respectively.

In this paper we only correct type I randoms because the type II and III randoms are insignificant in this study. The ratio of the number of randoms over the number of trues (randoms-to-trues ratio) for type I-III randoms were 4.9%, 0.1% and 0.003%, respectively in the simulated small animal scan with the reconstruction window [–1, 15] ns. The histogram of the lifetime measurements of true and random events is shown in Fig. 5. The total activity of the simulation scan was 2.78 MBq. The experimental scan has a comparable activity level of 3.7 MBq, leading to a negligible level of type II and III randoms as well. Thus, it is reasonable to neglect type II and III randoms for both simulation and experimental studies.

We opt to correct for type I randoms in the image domain. The two annihilation photons in type I randoms form a real coincident photon pair and can be mapped to an activity image $\gamma {{{\boldsymbol{x}}}}_{0}$, which is a scaled image of the standard activity image ${{{\boldsymbol{x}}}}_{0}$. ${{{\boldsymbol{x}}}}_{0}$ can be estimated by the standard PET acquisition of double coincidences without considering the prompt gammas and the scaling factor is given by $\gamma =\frac{\left[\# \; {{\rm{of}}}\; {{\rm{type}}}\, {{\rm{I}}}\,{{\rm{randoms}}}\right]}{\left[{{\#}}\; {{\rm{of}}}\; {{\rm{total}}}\; {{\rm{doubles}}}\right]}$. It is easy to obtain the number of doubles in the denominator. To estimate the number of type I randoms, we applied a correction window $[{t}_{{{\rm{c}}}1},{t}_{{{\rm{c}}}2}]$ that is shifted away from the true events. The events with lifetime measurements within such a window are predominantly type I randoms. As shown in Fig. 5, we set the correction window [–20, –5] ns in the simulation study. Since the lifetime measurements of type I randoms admit a uniform distribution, the number of type I randoms in the reconstruction window can be estimated by the events in the correction window. The location and width of the correction window is arbitrary as long as it excludes other types of events. Let $[{t}_{{{\rm{r}}}1},{t}_{{{\rm{r}}}2}]$ be the reconstruction window containing true events, which are set to -1 ns and 15 ns, respectively, in this study. The number of type I randoms is then estimated to be

14 $$\left[{{\#}}\; {{\rm{of}}}\; {{\rm{events}}}\; {{\rm{in}}}\; {{\rm{correction}}}\; {{\rm{window}}}\right]\times \frac{{t}_{{{\rm{r}}}2}-{t}_{{{\rm{r}}}1}}{{t}_{{{\rm{c}}}2}-{t}_{{{\rm{c}}}1}}$$

The forward projection model for the lifetime-weighted image becomes

15 $${z}_{i}={\sum }_{j=1}^{N}{H}_{{ij}}{\widetilde{w}}_{j}={\sum }_{j=1}^{N}{H}_{{ij}}\left({w}_{j}+\frac{{t}_{{{\rm{r}}}1}+{t}_{{{\rm{r}}}2}}{2}\gamma {x}_{{0}_{j}}\right)$$

where the factor $\frac{{t}_{{{\rm{r}}}1}+{t}_{{{\rm{r}}}2}}{2}$ accounts for the average lifetime measurement of the type I randoms on the support $[{t}_{{{\rm{r}}}1},{t}_{{{\rm{r}}}2}]$. Similarly, the forward projection model that dictates the activity data corresponding to ${{\boldsymbol{z}}}$ is

16 $${y}_{i}={\sum }_{j=1}^{N}{H}_{{ij}}{\widetilde{x}}_{j}={\sum }_{j=1}^{N}{H}_{{ij}}\left({x}_{j}+\gamma {x}_{{0}_{j}}\right)$$

We use original MLEM algorithm to obtain estimates for the type I-included activity image $\hat{\widetilde{{{\boldsymbol{x}}}}}$ and the total activity image ${\hat{{{\boldsymbol{x}}}}}_{0}$; and the modified MLEM algorithm to obtain an estimate for the lifetime-weighted image $\hat{\widetilde{{{\boldsymbol{w}}}}}$. With the estimated detector time shift $\hat{\mu }$, the final estimate for the lifetime image is

17 $${\hat{m}}_{j}=\frac{{\hat{\widetilde{w}}}_{j}-\frac{{t}_{{{\rm{r}}}1}+{t}_{{{\rm{r}}}2}}{2}\gamma {\hat{x}}_{{0}_{j}}}{{\hat{\widetilde{x}}}_{j}-\gamma {\hat{x}}_{{0}_{j}}}-\hat{\mu }$$

All the MLEM reconstruction was performed on a Dell computer with dual Intel Xeon E5-2630 v3 2.4 GHz CPUs, with acceleration achieved by OpenMP. For the simulation study, we performed normalization, attenuation correction and applied imaged-domain point spread function (PSF). The normalization and attenuation are the same as in the standard PET reconstruction for double coincidences. OSEM with two iterations and three subsets was used to reconstruct the activity, lifetime-weighted image and the standard activity image. The reconstruction FOV was 50 × 50 × 180 mm^3^ and the reconstruction voxel size was 0.8 × 0.8 × 1.6 mm^3^. For the experimental scan, we did not incorporate the factors for normalization and attenuation correction. Eight iterations were used for all the MLEM reconstructions. The reconstruction FOV was 128 × 128 × 64 mm^3^ and the voxel size was 1.0 × 1.0 × 1.0 mm^3^.

### Direct TOF-BP

Here we describe the direct TOF-BP method, which is used as the baseline method for comparison in this study. This method follows a similar pipeline as to the SIMPLE method—an activity image of triples, a lifetime-weighted image, and an activity image for randoms correction are obtained to form the lifetime image. But here these images are simply the product of the transposed TOF system matrix and the data. For instance, the lifetime-weighted image is calculated as

18 $${\hat{{{\boldsymbol{w}}}}}_{{{\rm{d}}}}={{{\boldsymbol{H}}}}^{{\prime} }{{\boldsymbol{z}}}$$

The three images ${\hat{{{\boldsymbol{x}}}}}_{{{\rm{d}}}}$, ${\hat{{{\boldsymbol{w}}}}}_{{{\rm{d}}}}$ and ${\hat{{{\boldsymbol{x}}}}}_{{0}_{{{\rm{d}}}}}$ are used to calculate the lifetime image by Eq. (17).

### Triple coincidence grouping

**Fig. 6:**
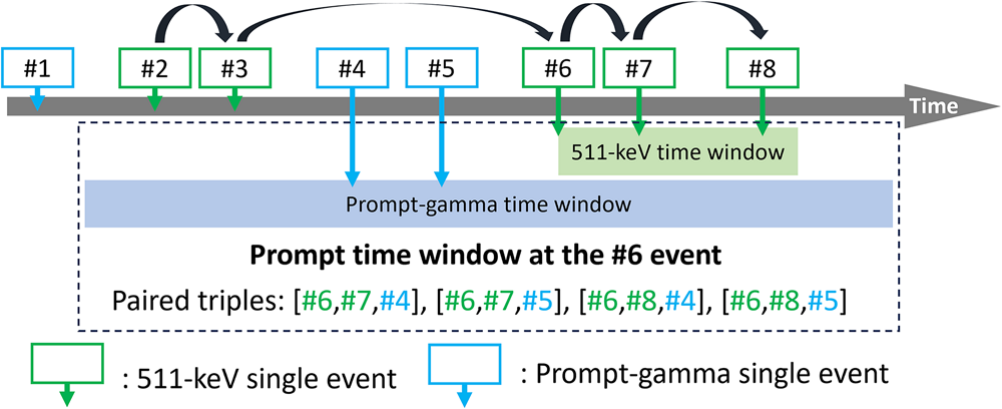
Illustration of the pairing process of prompt triple coincidences using the take-all-good policy. The prompt time window traverses every 511-keV single event and designates it as the reference event. This figure presents an example at the #6 event. A 511-keV time window is opened to the future to form double coincidences. For every double coincidence, the prompt-gamma event is found within the prompt-gamma time window. The width of prompt-gamma time window must include every event required by the reconstruction and randoms correction time window, $[{t}_{{{\rm{r}}}1},{t}_{{{\rm{r}}}2}]$ and $[{t}_{{{\rm{c}}}1},{t}_{{{\rm{c}}}2}]$ respectively.

In the simulation study, the triple coincidences were formed by grouping single events after the scan. First, the single events with energy within the 511-keV and prompt-gamma energy windows were sorted based on their temporal order. We iterated through each 511-keV single event and designated it as the reference event for a prompt time window which consists of two sub-windows—one for another 511-keV event and one for the prompt gamma. The 511-keV time window follows the standard PET approach. It searches for 511-keV events at a later time point. In contrast, the prompt-gamma time window spans from an earlier time point to a later time. This is because the prompt gammas are likely to be detected prior to the 511-keV photons. The take-all-good policy was employed to retrieve every eligible triple coincidence. This process is illustrated in Fig. 6.

In the experimental scan, the triple coincidences were reconstructed from double coincidences recorded online. Every double coincidence records crystal ID, energy, and detection time for its two singles. The detection time allows us to reconstruct a singles stream, with repetitive single events being discarded. Then we applied the prompt time window to group the triple coincidences. Note that although some single events were missed by the scanner if they cannot form a pair, all the singles within 15 ns to other singles are retained. Therefore, the lifetime spectrum from –15 ns to 15 ns is expected to be complete.

### Travel time correction

Since the prompt gamma and the annihilation photons are likely to travel different distances before being detected, it is necessary to estimate their emission times for an accurate lifetime measurement. First, an annihilation point is inferred as the most likely position along the LOR using the TOF information of the two 511-keV photons. Then the travel time is calculated as the flying time between the annihilation point and the detection point in the detector ring. The estimated travel time is subsequently subtracted from the detection time to estimate the emission time. The emission time of annihilation photons is computed as ${t}_{511}=({t}_{1}+{t}_{2}-\frac{{d}_{{{\rm{lor}}}}}{c})/2$, where ${t}_{1}$ and ${t}_{2}$ are the detection times of the two 511-keV photons, ${d}_{{{\rm{lor}}}}$ is the distance between two detectors, and $c$ is the speed of light. Finally, the lifetime measurement of each PLI event is computed as $\tau ={t}_{511}-{t}_{{{\rm{PG}}}}$, where ${t}_{{PG}}$ is the estimated time of the prompt gamma emission. Note that this correction is only performed for each lifetime measurement, whereas the TOF information used in the activity image reconstruction is unaltered.

### Reporting summary

Further information on research design is available in the Nature Portfolio Reporting Summary linked to this article.

## Supplementary information

Reporting Summary

## Data Availability

The list-mode PET data of the simulation and the experimental studies, and the reconstructed images are available at https://qilab.bme.ucdavis.edu/simple-paper.

## Code availability

The code of SIMPLE reconstruction and simulation data processing can be accessed via Github at https://github.com/Acurbbb/SIMPLE.
